# Supplementary material for: Effects of high-intensity interval training on physical morphology, cardiorespiratory fitness and metabolic risk factors of cardiovascular disease in children and adolescents: A systematic review and meta-analysis
Source: PLoS One. 2023 May 11;18(5):e0271845. doi: 10.1371/journal.pone.0271845 (PMC10174557; doi:10.1371/journal.pone.0271845)
Supplement: S1 Table — (DOCX) [file pone.0271845.s001.docx]

**S1 Table** **Search Strategy.**

**Pubmed:**

| **Items** | **Search Terms** | **Search Results** |
| --- | --- | --- |
| #1 | Adolescent [MeSH Terms] | 2148189 |
| #2 | (((((Adolescence [Title/Abstract]) OR (Teens [Title/Abstract])) OR (Teenagers [Title/Abstract])) OR (Youths [Title/Abstract])) OR (Female Adolescents [Title/Abstract])) OR (Male Adolescents [Title/Abstract]) | 113579 |
| #3 | #1 or #2 | 2175701 |
| #4 | High-Intensity Interval Training [MeSH Terms] | 1636 |
| #5 | ((((High-Intensity Interval [Title/Abstract]) OR (High-Intensity Intermittent [Title/Abstract])) OR (High-Intensity Intermittent Exercises [Title/Abstract])) OR (Sprint Interval Trainings [Title/Abstract])) OR (HIIT[Title/Abstract]) | 3458 |
| #6 | #4 or #5 | 3849 |
| #7 | randomized controlled trial [MeSH Terms] | 155174 |
| #8 | (((RCT[Title/Abstract]) OR (Randomized [Title/Abstract])) OR (Randomized Clinical [Title/Abstract])) OR (Controlled Clinical Trials [Title/Abstract]) | 611902 |
| #9 | #7 or #8 | 694148 |
| #10 | #9 AND #6 AND #3 | 92 |

**Cochrane Library:**

| **Items** | **Search Terms** | **Search Results** |
| --- | --- | --- |
| #1 | MeSH descriptor: [Adolescent] explode all trees | 108630 |
| #2 | (Adolescence): ti,ab,kw OR (Teens):ti,ab,kw OR (Teenagers):ti,ab,kw OR (Youths):ti,ab,kw | 8461 |
| #3 | #1 OR #2 | 114958 |
| #4 | MeSH descriptor: [High-Intensity Interval Training] explode all trees | 588 |
| #5 | (High-Intensity Interval): ti,ab,kw OR (High-Intensity Intermittent):ti,ab,kw OR (High-Intensity Intermittent Exercises):ti,ab,kw OR (Sprint Interval Trainings):ti,ab,kw OR (HIIT):ti,ab,kw | 3328 |
| #6 | #4 OR #5 | 3328 |
| #7 | MeSH descriptor: [Randomized Controlled Trial] explode all trees | 119 |
| #8 | (RCT): ti,ab,kw OR (Randomized):ti,ab,kw OR (Randomized Clinical):ti,ab,kw OR (Controlled Clinical Trials):ti,ab,kw | 1002643 |
| #9 | #5 or #6 | 1002643 |
| #10 | #7 and #8 and #9 | 143 |

**Embase**:

| **Items** | **Search Terms** | **Search Results** |
| --- | --- | --- |
| #1 | 'adolescent'/exp OR 'adolescent' OR 'adolescence'/exp OR 'adolescence' OR 'teens' OR 'teenagers' OR 'youths' OR 'female adolescents' OR 'male adolescents' | 1971546 |
| #2 | 'high-intensity interval training'/exp OR 'high-intensity interval training' OR 'high-intensity interval' OR 'high-intensity intermittent' OR 'high-intensity intermittent exercises' OR 'sprint interval trainings' OR 'hiit'/exp OR 'hiit' | 5171 |
| #3 | 'randomized controlled trial' OR 'rct' OR 'randomized' OR 'randomized clinical' OR 'controlled clinical trials' | 1276675 |
| #4 |  | 151 |

**Web of science:**

| **Items** | **Search Terms** | **Search Results** |
| --- | --- | --- |
| #1(participents) | TS=(adolescent OR Adolescence OR Teens OR Teenagers OR Youths OR Female Adolescents OR Male Adolescents) | 2837751 |
| #2(intervention) | TS=(High-Intensity Interval Training OR High-Intensity Interval OR High-Intensity Intermittent OR High-Intensity Intermittent Exercises OR Sprint Interval Trainings OR hiit) | 5171 |
| #3(study) | TS=(randomized controlled trial OR RCT OR Randomized OR Randomized Clinical OR Controlled Clinical Trials) | 1435301 |
| #4 | #1 AND #2 AND #3 | 345 |

**Science Direct (2015-now):**

| **Search Terms** | **Search Results** |
| --- | --- |
| (Adolescent OR Teens OR Youths) AND (High-Intensity Interval Training OR High-Intensity Intermittent Exercises OR Sprint Interval Trainings) AND (randomized controlled trial OR RCT OR Controlled Clinical Trials) | 2948 |

**CNKI：**

| **Search Terms** | **Search Results** |
| --- | --- |
| (SU=''青少年" OR SU="儿童'' OR SU="女青年" OR SU="男青年" OR SU="男儿童" OR SU="女儿童" OR SU="男运动员" OR SU="女运动员") AND (SU="高强度间歇训练" OR SU="冲刺间歇训练" OR SU="高强度间歇跳绳训练" OR SU="间歇训练" OR SU="有氧间歇训练" OR SU="无氧间歇训练" OR SU="长间歇训练" OR SU="短间歇训练" OR SU="高强度有氧间歇训练") AND (SU="随机对照试验" OR SU="随机化" OR SU="随机分配" OR SU="单盲" OR SU="双盲" OR SU="对照" OR SU="对照试验") | 40 |

**Wanfang：**

| **Search Terms** | **Search Results** |
| --- | --- |
| (主题:"青少年" or 主题:"儿童" or 主题:"女青年" or 主题:"男青年" or 主题:"男儿童" or 主题:"女儿童" or 主题:"男运动员" or 主题:"女运动员") and (主题:"高强度间歇训练" or 主题:"冲刺间歇训练" or 主题:"高强度间歇跳绳训练" or 主题:"间歇训练" or 主题:"有氧间歇训练" or 主题:"无氧间歇训练" or 主题:"长间歇训练" or 主题:"短间歇训练" or 主题:"高强度有氧间歇训练") and (主题:"双盲" or 主题:"单盲" or 主题:"对照" or 主题:"随机化" or 主题:"随机对照试验" or 主题:"随机分配" or 主题:"对照实验") | 15 |

**VIP：**

| **Search Terms** | **Search Results** |
| --- | --- |
| (M=青少年 OR M=儿童 OR M=女青年 OR M=男青年 OR M=男儿童 OR M=女儿童 OR M=男运动员 OR M=女运动员) AND (M=高强度间歇训练 OR M=冲刺间歇训练 OR M=高强度间歇跳绳训练 OR M=间歇训练 OR M=有氧间歇训练 OR M=无氧间歇训练 OR M=长间歇训练 OR M=短间歇训练 OR M=高强度有氧间歇训练) AND (M=随机对照试验 OR M=随机化 OR M=随机分配 OR M=单盲 OR M=双盲 OR M=对照 OR M=对照实验) | 0 |
