# Supplementary material for: Effects of high-intensity interval training on physical morphology, cardiorespiratory fitness and metabolic risk factors of cardiovascular disease in children and adolescents: A systematic review and meta-analysis
Source: PLoS One. 2023 May 11;18(5):e0271845. doi: 10.1371/journal.pone.0271845 (PMC10174557; doi:10.1371/journal.pone.0271845)
Supplement: S2 Table — (DOCX) [file pone.0271845.s002.docx]

**S2 Table: Excluded Studies and Reason for Exclusion.**

| **N** | **First Author（Year）** | **Title** | **Reason for exclusion** |
| --- | --- | --- | --- |
| 1 | T. Matsuo (2015) [1] | Effect of aerobic exercise training followed by a low-calorie diet on metabolic syndrome risk factors in men | Age out of range |
| 2 | G. Baquet (2001) [2] | High-Intensity Aerobic Training During a 10 Week One-Hour Physical Education Cycle: Effects on Physical Fitness of Adolescents Aged 11 to 16 | No result measurement applicable |
| 3 | André Filipe Paulino da Silva Bento (2021) [3] | High-intensity interval training in high-school physical education classes:  Study protocol for a randomized controlled trial | No result measurement applicable |
| 4 | ORL Farley (2016) [4] | Five Weeks of Sprint and High Intensity Interval Training Improves Paddling Performance in Adolescent Surfers | There is no blank control |
| 5 | Vivian Bertoni Xavier (2020) [5] | Combined aerobic and resistance training improves respiratory and exercise  outcomes more than aerobic training in adolescents with idiopathic scoliosis: a randomized trial | There is no blank control |
| 6 | Leskinen (2011) [6] | [This is an electronic reprint of the original article. This reprint may differ from the original in pagination and typographic detail.](http://research.aalto.fi/files/26124425/paper7.pdf) | No result measurement applicable |
| 7 | Víctor Hugo Arboleda-Serna (2019) [7] | Effects of high-intensity interval training compared to moderate-intensity continuous training on maximal oxygen consumption and blood pressure  in healthy men: A randomized controlled trial | Age out of range |
| 8 | TA Bakken (2013) [8] | Effects of block periodization training versus traditional periodization training in trained cross-country skiers | No result measurement applicable |

**Reference:**

1.Matsuo T , So R , Shimojo N , et al. Effect of aerobic exercise training followed by a low-calorie diet on metabolic syndrome risk factors in men[J]. Nutrition, Metabolism and Cardiovascular Diseases, 2015.

2.Karabay Z A , T Özkan, A Koç, et al. High-Intensity Aerobic Training During a 10 Week One-Hour Physical Education Cycle: Effects on Physical Fitness of Adolescents Aged 11 to 16[J]. International Journal of Sports Medicine, 2001, 22(04):295-300.

3.André Filipe Paulino da Silva Bento a, Lcp B , Amdmr A . High-intensity interval training in high-school physical education classes: Study protocol for a randomized controlled trial - ScienceDirect.

4.Farley O , Secomb J L , Parsonage J , et al. Five Weeks Of Sprint And High Intensity Interval Training Improves Paddling Performance In Adolecent Surfers[J]. Journal of Strength and Conditioning Research, 2016:1.

5.Vbx A , Oa A , Bruno Derwood Mills Costa de Carvalho a, et al. Combined aerobic and resistance training improves respiratory and exercise outcomes more than aerobic training in adolescents with idiopathic scoliosis: arandomised trial[J]. Journal of Physiotherapy, 2020, 66( 1):33-38.

6.Leskinen, P Hyvönen, Tuominen E , et al. This is an electronic reprint of the original article. This reprint may differ from the original in pagination and typographic detail.

7.Víctor, Hugo, Arboleda-Serna, et al. Effects of high-intensity interval training compared to moderate-intensity continuous training on maximal oxygen consumption and blood pressure in healthy men: A randomized controlled trial[J]. Biomedica : revista del Instituto Nacional de Salud, 2019, 39(3):524-536.

8.Bakken T A . Effects of block periodization training versus traditional periodization training in trained cross country skiers[J]. Department of Sport & Health Sciences, 2013.
