# Supplementary material for: Effects of high-intensity interval training on physical morphology, cardiorespiratory fitness and metabolic risk factors of cardiovascular disease in children and adolescents: A systematic review and meta-analysis
Source: PLoS One. 2023 May 11;18(5):e0271845. doi: 10.1371/journal.pone.0271845 (PMC10174557; doi:10.1371/journal.pone.0271845)
Supplement: S3 Table — (DOCX) [file pone.0271845.s003.docx]

**S3 Table Detailed table of basic characteristics of included studies.**

| **Group**  **Characteristics** |  | **HIIT Group** | **Control Group** | **Total** |
| --- | --- | --- | --- | --- |
| **Gender**  **(Unit: person)** | Boys | 741(42.37%) | 424(34.03%) | 1165(38.90%) |
|  | Girls | 686(39.22%) | 470(37.72%) | 1156(38.60%) |
|  | Only Girls | 163(9.32%) | 163(13.08%) | 326(10.88%) |
|  | Only Boys | 107(6.12%) | 107(8.59%) | 214(7.15%) |
|  | Gender-neutral | 322(18.41%) | 374(30.02%) | 696(23.24%) |
|  | Total | 1749 | 1246 | 2995 |
| **Weight (Overweight/obesity)**  **(Unit: person)** | Boys | 84(37.50%） | 70(32.71%) | 154(35.16%) |
|  | Girls | 92(41.07%) | 84(39.25%) | 176(40.18%) |
|  | Only Girls | 81(31.16%) | 75(35.05%) | 156(35.62%) |
|  | Only Boys | 56(25%) | 107(8.59%) | 163(37.21%) |
|  | Gender-neutral | 109(27.81%) | 60(28.04%) | 169(38.58%) |
|  | Total | 224 | 214 | 438 |
| **Follow-up Time (Unit: term)**  **Average Weeks：10.99 Weeks** | 1.57Weeks(10Days)/40Weeks | 1(2.13%) /1(2.13%) | | |
|  | 8 Weeks | 14（29.79%) | | |
|  | 12 Weeks | 10（21.82%） | | |
| **Exercise Frequency**  **(Unit: term)** | Twice/ Weeks | 18(40.00%) | | |
|  | 3 times/ Weeks | 21(46.67%) | | |
|  | 4 times / Weeks | 2(4.44%) | | |
|  | 5 times / Weeks | 1(2.22%) | | |
|  | Increase exercise frequency and not explicitly described | 5(11.11%) | | |
|  | Total | 45 | | |
| **Exercise Mode (Unit: term)** | Power bike | 3(6.38%) | | |
|  | Running (indoor/outdoor) | 19(40.43%) | | |
|  | Else | 21(44.68%) | | |
|  | Not explicitly described | 5(8.51%0 | | |
| **Age（5～19）** | Children (5~15) | 2328(77.73%) | | |
|  | Adolescents(＞15) | 667(22.27%) | | |
| **Participants (Unit: person)** | Overweight and Obese | 438(14.62%) | | |
|  | Athletes | 62(2.07%) | | |
|  | Sick children and adolescents | 261(8.71%) | | |
| **Medical Supervision**  **(Unit: term)** | With medical supervision | 26 | | |
|  | Without medical supervision | 0 | | |
|  | Not explicitly described | 21 | | |
