# Supplementary material for: Effects of high-intensity interval training on physical morphology, cardiorespiratory fitness and metabolic risk factors of cardiovascular disease in children and adolescents: A systematic review and meta-analysis
Source: PLoS One. 2023 May 11;18(5):e0271845. doi: 10.1371/journal.pone.0271845 (PMC10174557; doi:10.1371/journal.pone.0271845)
Supplement: S4 Table — (DOCX) [file pone.0271845.s004.docx]

**S4 Table Measurement method(s) and distribution of outcome indicators.**

| Sorts | Outcomes | Measurement method(s) |
| --- | --- | --- |
| Physical morphology | BMI  (37) | digital scale measurements [38, 65-72, 74, 75, 77-82, 84-90, 92, 93, 95-97, 100, 101, 103-106, 108] |
|  |  | not explicitly described [65] |
|  | BF%  (20) | multi-frequency bioelectrical impedance technology body composition analyzer [66, 70, 72, 77, 82, 95-99, 105] |
|  |  | skinfold thickness calculation method [63, 64, 67, 73, 74, 103] |
|  |  | Dual Energy X-ray Absorptiometry (DEXA)[38, 87] |
|  |  | not explicitly described [68] |
|  | WC  (16) | tape measure [66, 78, 87, 89, 90, 97, 98, 103-105, 108] |
|  |  | not explicitly described [38, 82, 95, 99, 100] |
| CRF | VO_2max_  (19) | power bikes, treadmills and other external equipment  [67, 72, 76, 82, 87, 90, 91, 95] |
|  |  | portable cardiopulmonary function tester [38, 63, 65, 97, 99] |
|  |  | 20m turn-back running method [79, 80, 85, 89, 94, 104] |
|  | SBP/DBP  (14/14) | automatic blood pressure tester [38, 83, 89, 104, 105] |
|  |  | manual blood pressure tester [66, 70, 95, 98] |
|  |  | not explicitly described [68, 72, 75, 100] |
|  | HR_max_  (11) | heart rate monitor [63, 65, 67, 70, 82, 86, 91, 92, 95, 98, 102] |
|  |  | not explicitly described [84] |
| Cardiometabolic indexes | TC/TG/HDL-C  (8/8/7) | enzymatic methods [95, 97] |
|  |  | standard colorimetric assays [90] |
|  |  | spectrophotometry [89] |
|  |  | portable measuring instrument [100] |
|  |  | not explicitly described [68] |
|  | LDL-C  (7) | Friedewald formula [63, 73, 80, 89, 92] |
|  |  | portable measuring instrument [95] |
|  |  | not explicitly described [97] |
