# Supplementary figures and images for: Effects of high-intensity interval training on physical morphology, cardiorespiratory fitness and metabolic risk factors of cardiovascular disease in children and adolescents: A systematic review and meta-analysis
Source: PLoS One. 2023 May 11;18(5):e0271845. doi: 10.1371/journal.pone.0271845 (PMC10174557; doi:10.1371/journal.pone.0271845)

**S1 Fig Sensitivity analysis of BMI.**


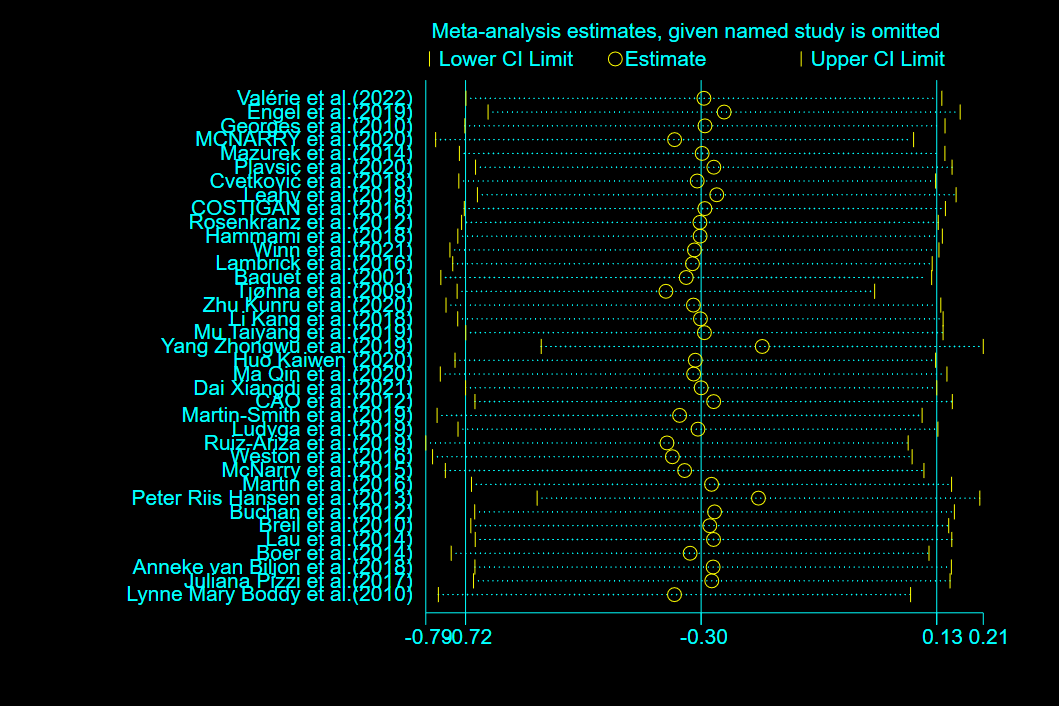

Supplement: S1 Fig — (DOCX) [file pone.0271845.s005.docx]

**S2 Fig Sensitivity analysis of BF%.**


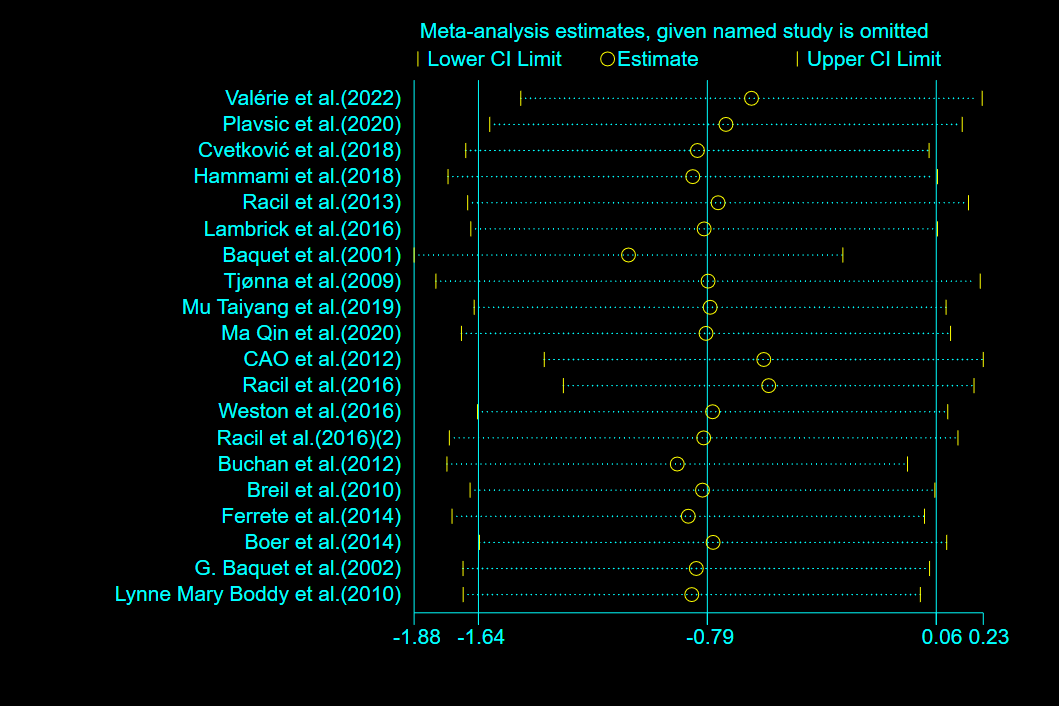

Supplement: S2 Fig — (DOCX) [file pone.0271845.s006.docx]

**S3 Fig** **Sensitivity analysis of WC.**


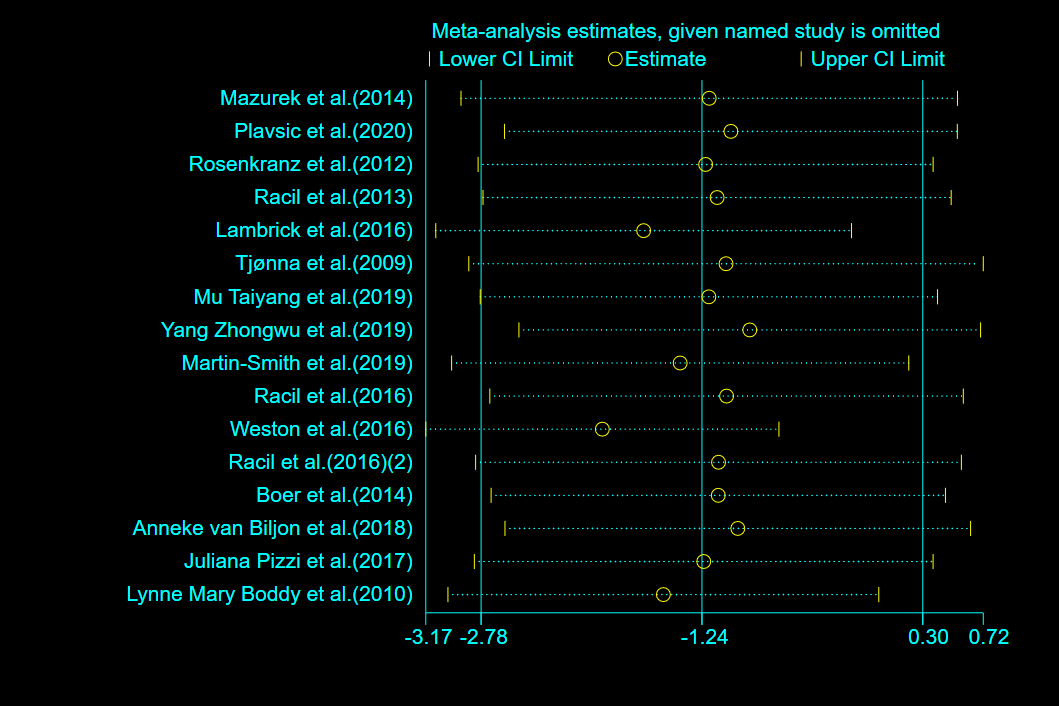

Supplement: S3 Fig — (DOCX) [file pone.0271845.s007.docx]

**S4 Fig** **Sensitivity analysis of TC.**


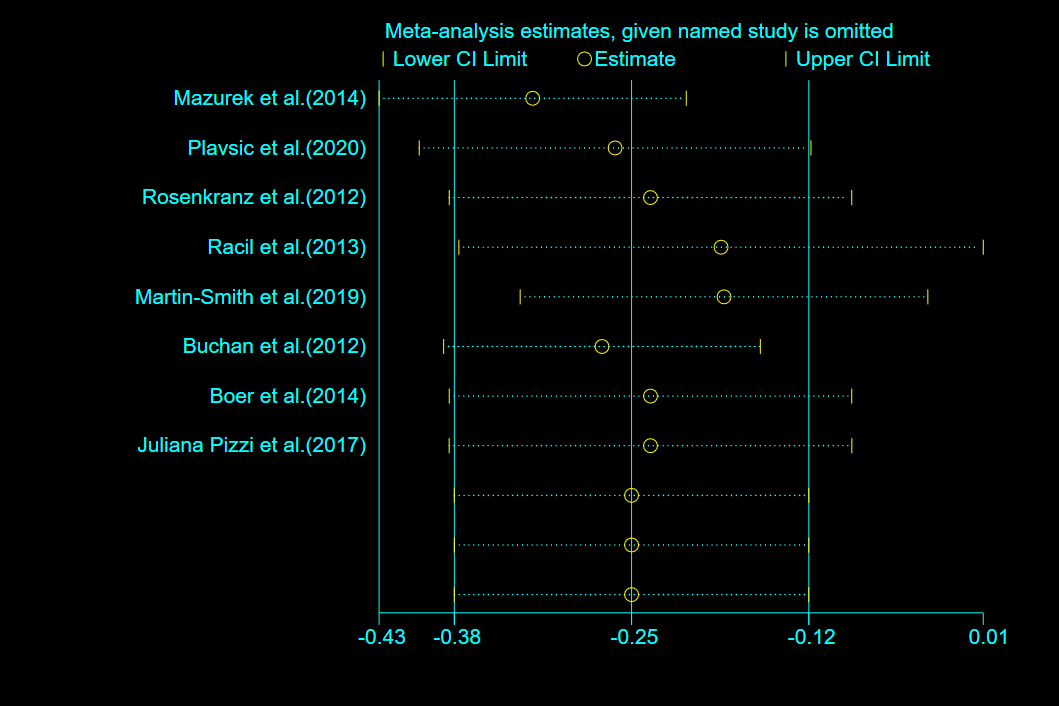

Supplement: S4 Fig — (DOCX) [file pone.0271845.s008.docx]

**S5 Fig** **Sensitivity analysis of TG.**


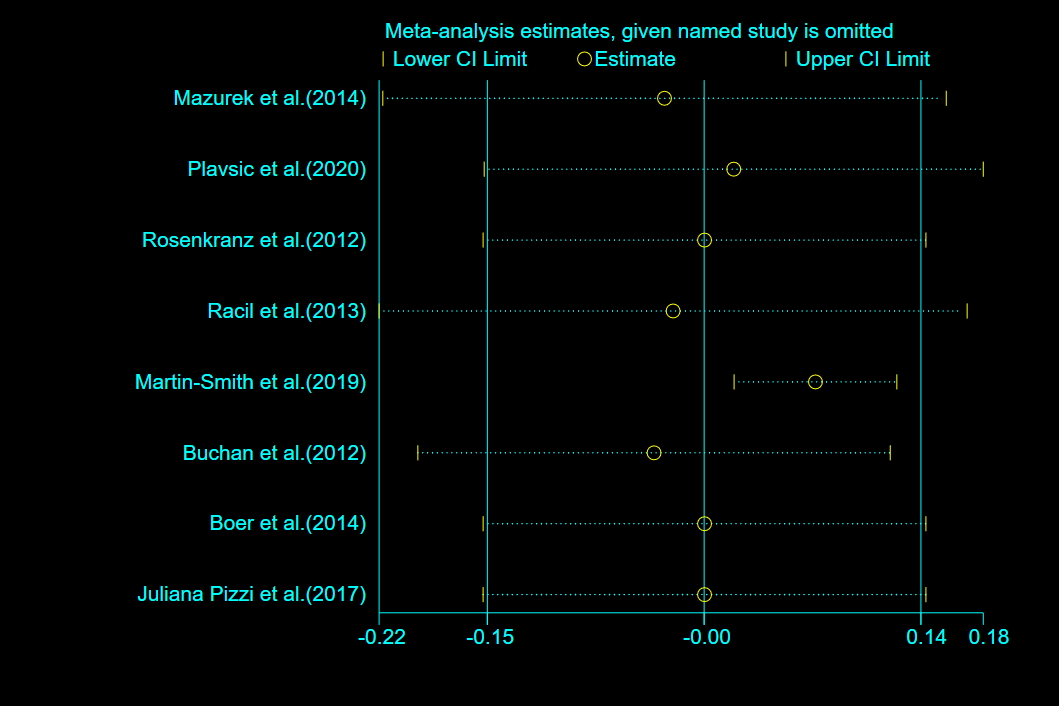

Supplement: S5 Fig — (DOCX) [file pone.0271845.s009.docx]

**S6 Fig** **Sensitivity analysis of HDL-C.**


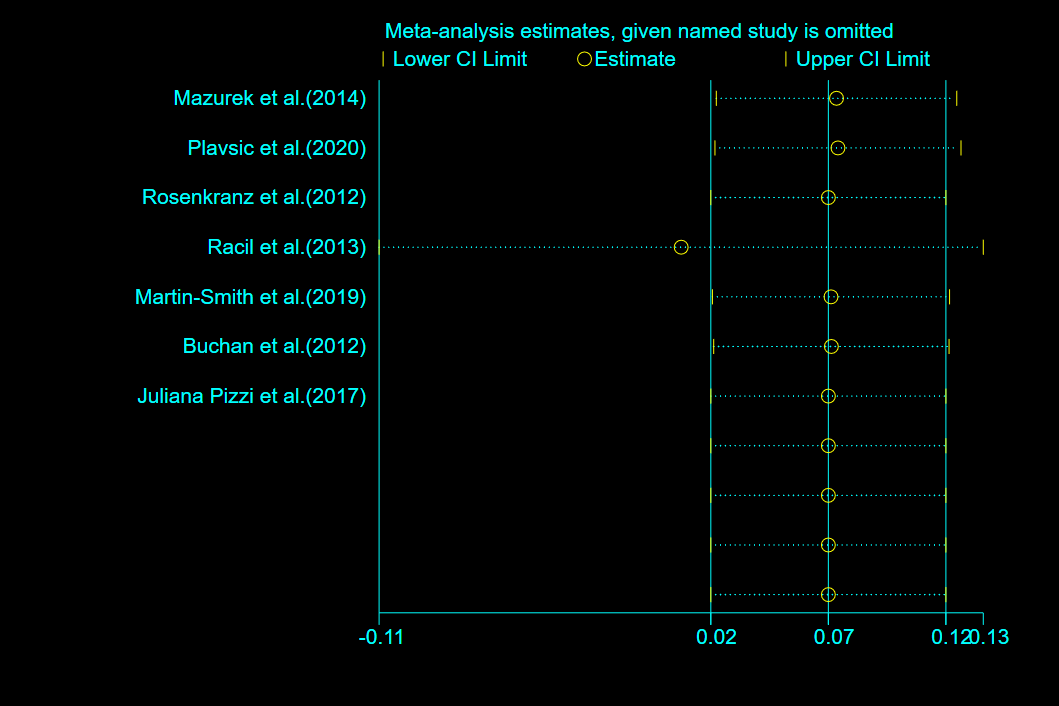

Supplement: S6 Fig — (DOCX) [file pone.0271845.s010.docx]

**S7 Fig** **Sensitivity analysis of LDL-C.**


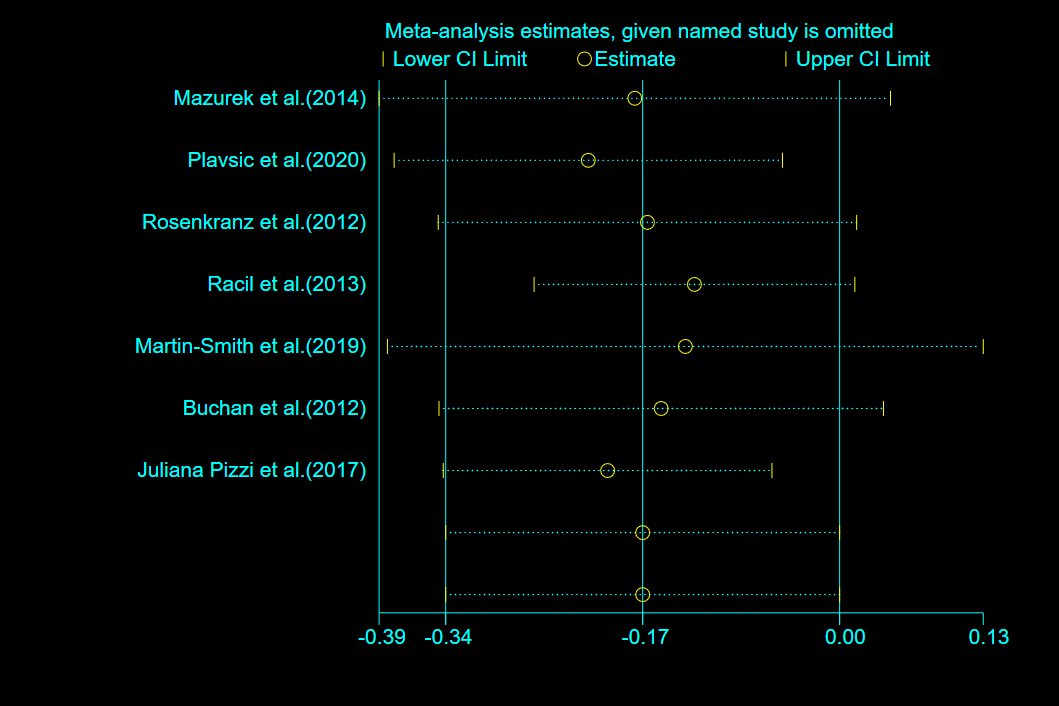

Supplement: S7 Fig — (DOCX) [file pone.0271845.s011.docx]

**S8 Fig** **Sensitivity analysis of VO2max.**


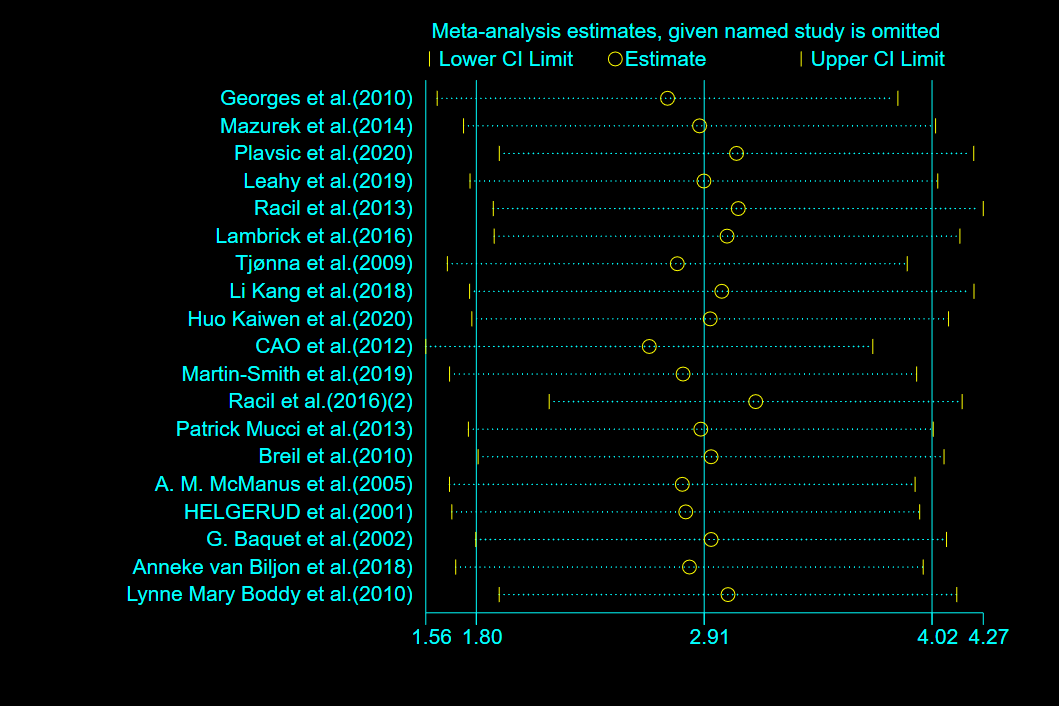

Supplement: S8 Fig — (DOCX) [file pone.0271845.s012.docx]

**S9 Fig Sensitivity analysis of SBP.**


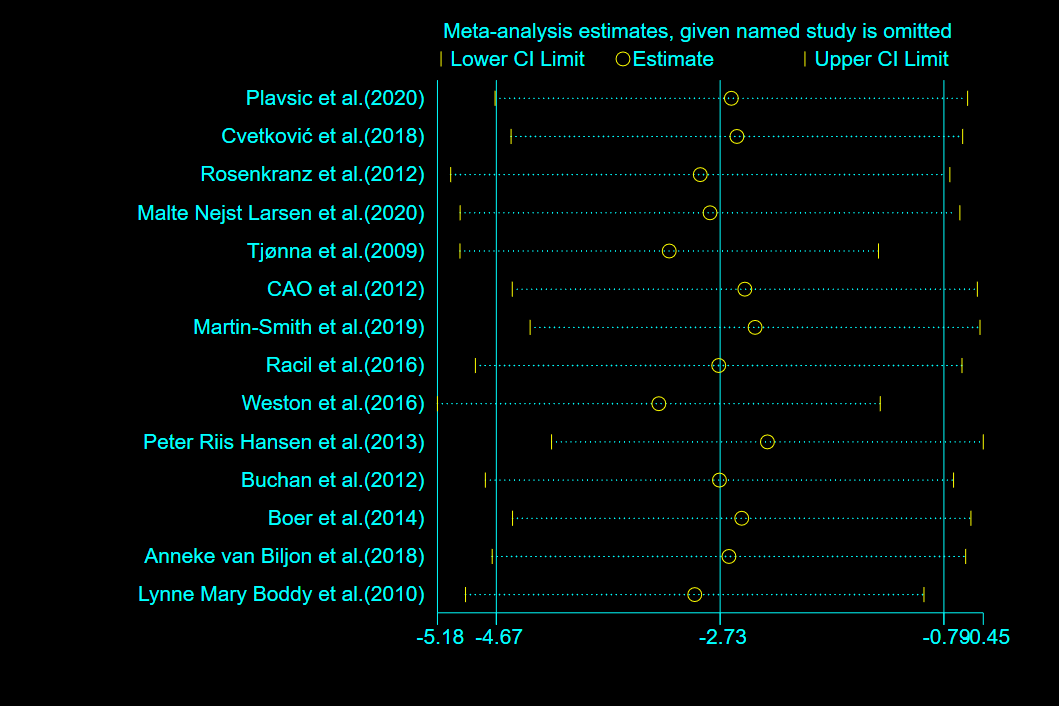

Supplement: S9 Fig — (DOCX) [file pone.0271845.s013.docx]

**S10 Fig Sensitivity analysis of DBP.**


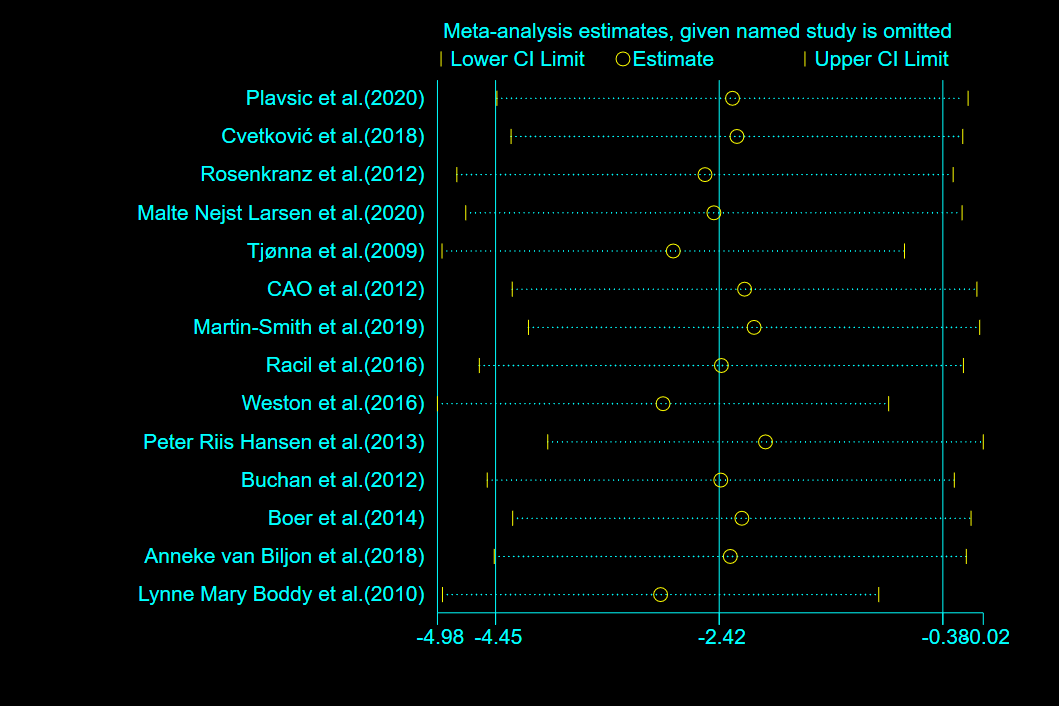

Supplement: S10 Fig — (DOCX) [file pone.0271845.s014.docx]

**S11 Fig** **Sensitivity analysis of HRmax.**


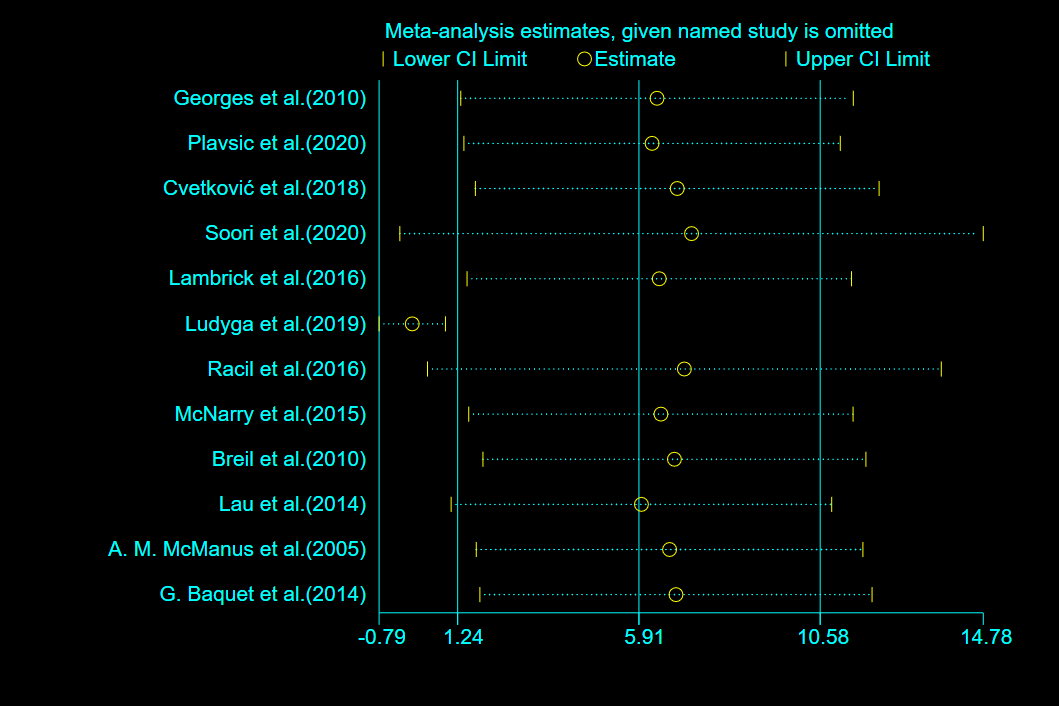

Supplement: S11 Fig — (DOCX) [file pone.0271845.s015.docx]

**S12 Fig Subgroup analysis of age in children and adolescents with VO2max in HIIT and**

**control group.**


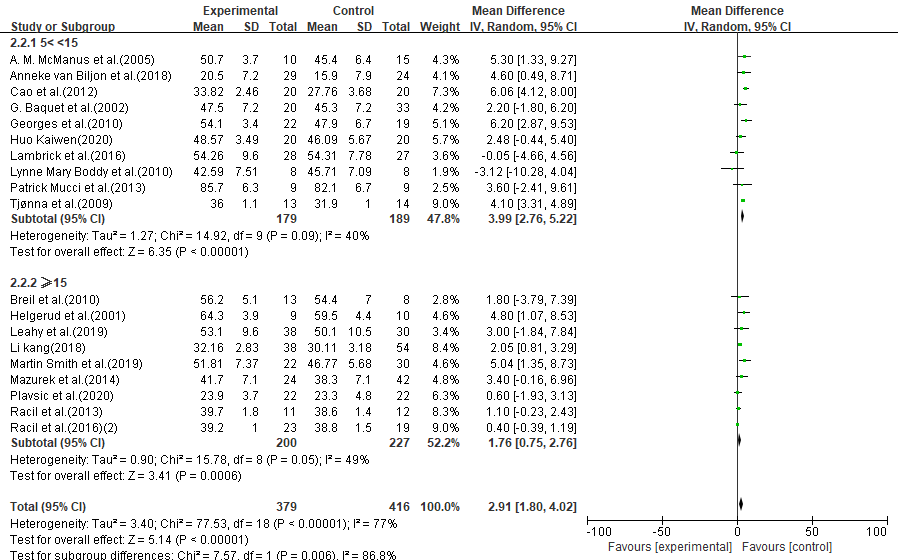

Supplement: S12 Fig — (DOCX) [file pone.0271845.s016.docx]

**S16 Fig Subgroup analysis of age in children and adolescents with SBP in HIIT and control group.**


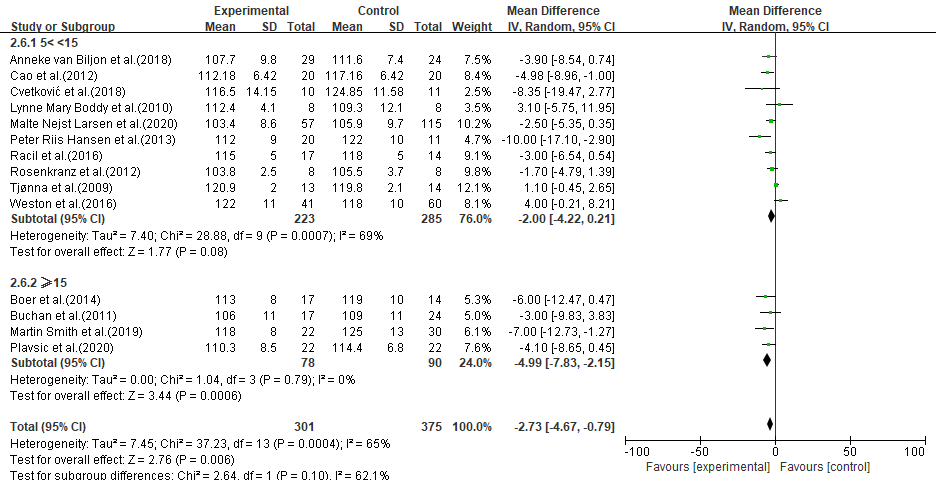

Supplement: S16 Fig — (DOCX) [file pone.0271845.s020.docx]

**S20 Fig Subgroup analysis of age in children and adolescents with DBP in HIIT and control group.**


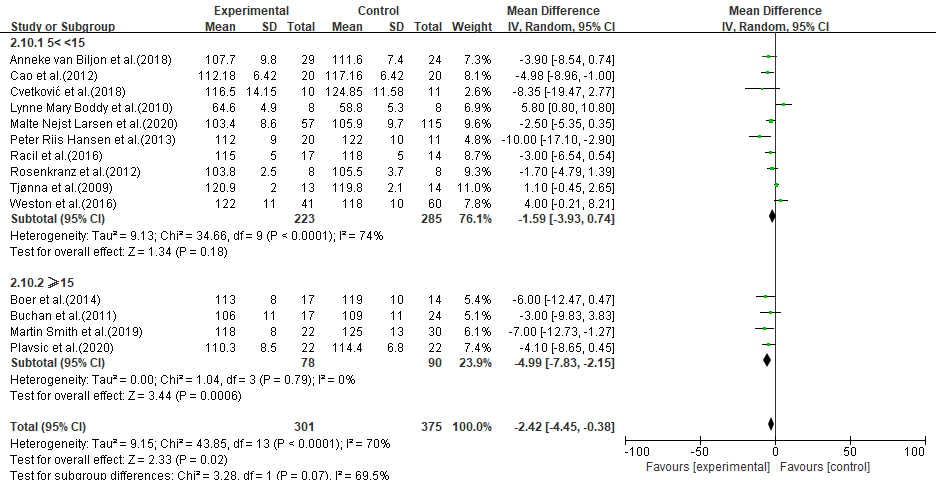

Supplement: S20 Fig — (DOCX) [file pone.0271845.s024.docx]

**S24 Fig Subgroup analysis of age in children and adolescents with HRmax in HIIT and control group.**


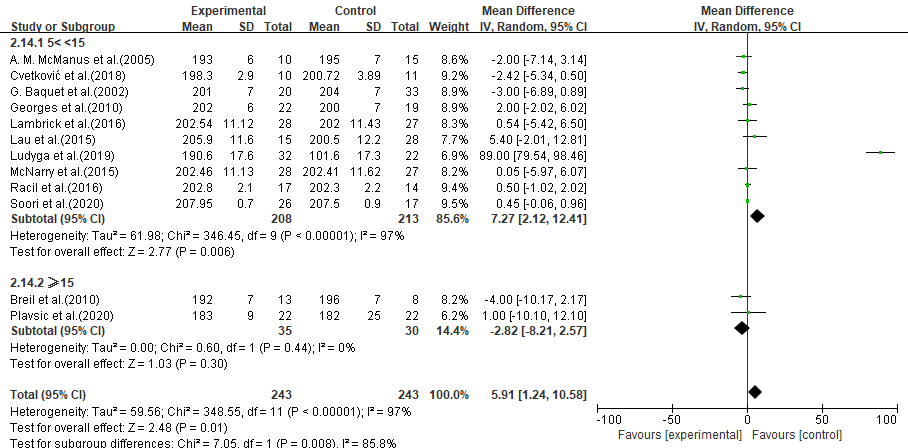

Supplement: S24 Fig — (DOCX) [file pone.0271845.s028.docx]
