# Supplementary material for: Effects of high-intensity interval training on physical morphology, cardiorespiratory fitness and metabolic risk factors of cardiovascular disease in children and adolescents: A systematic review and meta-analysis
Source: PLoS One. 2023 May 11;18(5):e0271845. doi: 10.1371/journal.pone.0271845 (PMC10174557; doi:10.1371/journal.pone.0271845)

**S18 Fig Subgroup analysis of intervention time in children and adolescents with SBP in HIIT and control group.**


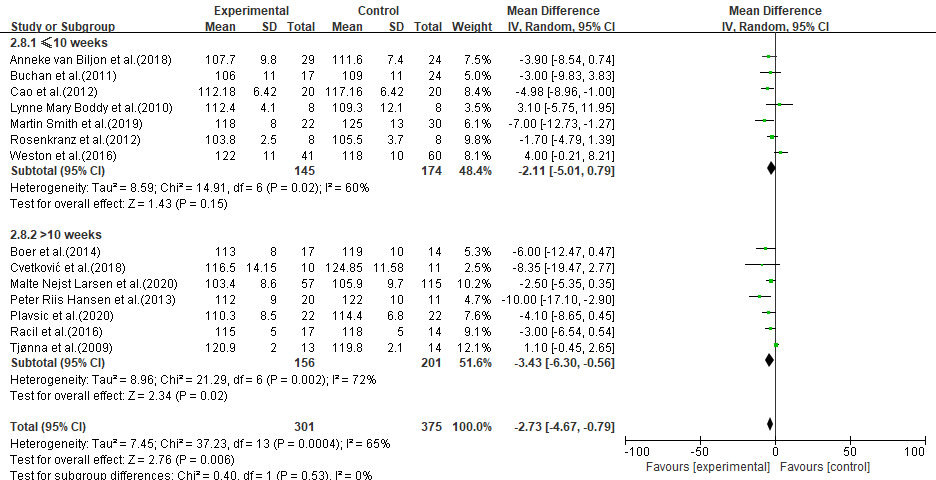

Supplement: S18 Fig — (DOCX) [file pone.0271845.s022.docx]
