# Supplementary material for: Effects of high-intensity interval training on physical morphology, cardiorespiratory fitness and metabolic risk factors of cardiovascular disease in children and adolescents: A systematic review and meta-analysis
Source: PLoS One. 2023 May 11;18(5):e0271845. doi: 10.1371/journal.pone.0271845 (PMC10174557; doi:10.1371/journal.pone.0271845)
Supplement: Attachment — Submitted filename: Response to Reviewers.docx [file pone.0271845.s033.docx]

**Response to Reviewers**

Deare editor and reviewers，

Thank you for offering us an opportunity to improve the quality of our submission manuscript (PONE-D-22-19095). We appreciated very much the reviewers’ constructive and insightful comments. In this revision, we have addressed all these comments and suggestions. We hope that the revised manuscript has now met the publication standard of your journal. We highlighted all the revisions in red colour.

Firstly, the following changes have been made to meet the additional requirements of this journal:

1.We have corrected incorrect words and grammar to meet the rules of standard English writing wherever possible.

2. Support & Fund Information：Teaching Reform and Innovation Project for Higher Education Institutions of Shanxi Provincial Education Department (No.J2021967)、Teaching Reform Innovation Project of Fenyang College of Shanxi Medical University (No.FJ202013) and 2022 Research Project of Fenyang College of Shanxi Medical University (No.2022A01)。FJ202013 and 2022A01 provide platform resources in terms of data collection and analysis.J2021967 provided constructive advice on publication decisions and manuscript preparation.

3. Table 2 “Basic features of the included studies” and Table 2 “Three-line table of Egger’s Publishing Bias” in the original manuscript were incorrectly converted by the software when converting from docx format to pdf format, resulting in an error in the table names.

**Response to reviewer**

**Is the manuscript technically sound, and do the data support the conclusions?**

**The manuscript must describe a technically sound piece of scientific research with data that supports the conclusions. Experiments must have been conducted rigorously, with appropriate controls, replication, and sample sizes. The conclusions must be drawn appropriately based on the data presented.**

**Reviewer #1: Partly**

**Reviewer #2: Partly**

**Response**：Based on the reviewers' suggestions and comments, we have re-run the statistical analysis of the data. In the discussion section, we start with the results of the available evidence, re-analyse potential sources of heterogeneity and discuss them in detail after identifying their significant sources of heterogeneity and compare them with previous studies (e.g. intervention methods, findings), especially the parts with different or controversial findings. Given that there are still some limitations to this article, the specific exercise interventions we recommend are only an objective suggestion (see the discussion section for more details).

**Reviewer #1: The title is not correct, erroneously suggesting a beneficial effect of HIIT on all cardiometabolic indicators.**

**Numerous editorial errors indicating careless preparation of the manuscript. Conclusions not entirely correct.**

**Response**：The corrected title is “Effects of high-intensity interval training on physical morphology, cardiopulmonary fitness and metabolic risk indicators of cardiovascular disease in children and adolescents: a systematic review and meta-analysis.”

**In the opinion of the reviewer, the age range of the analysed group is too wide for a single metanalysis. This type of review should be carried out for at least three age groups, e.g., preschool and early school age children, high school students and young adults.**

**Response**：Our division into age groups was based on the actual situation of the included studies. If the studies were divided into at least three groups as suggested by the reviewers, there would be only four relevant studies in which the age stage of the study population belonged to young people, which is a smaller sample size than the other groups and insufficient to support the results of a robust subgroup analysis. Therefore the subgroups in this subgroup analysis were grouped according to 5- 15 years and ≥15 years. Although the age range is too wide, more robust results can be obtained.

**Reviewer #2: Systematic review title should have to be checked if others are running it through PROSPERO. Hence, this should be explained under methods part.**

**Response：**We were registered with PROSPERO under the registration number 10.37766/inplasy 2022.10.0092.

**Line 219 "This study is the first to systematically..." are you sure for this? (what about this study? DOI: 10.1101/2022.07.11.22277515)**

**Response**：This article (DOI: 10.1101/2022.07.11.22277515) is a preprint version of the manuscript that we published in medRxiv (<https://medrxiv.org/cgi/content/short/2022.07.11.22277515v1>).

**PLOS authors have the option to publish the peer review history of their article (what does this mean?). If published, this will include your full peer review and any attached files.**

**If you choose “no”, your identity will remain anonymous but your review may still be made public.**

**Do you want your identity to be public for this peer review? For information about this choice, including consent withdrawal, please see our Privacy Policy.**

**Reviewer #1: No**

**Reviewer #2: No**

**Response：**Consent for my identity to be made public in peer review.

I hope you are satisfied with our changes. If you have any questions, please be sure to let me know, I will reply to you as soon as possible after receiving comments.

Declaration: The manuscript (in whole or part) has not been submitted or published in other journals at the same time. All authors have read and agreed to the content of the manuscript, and there is no potential conflict of interest.

Thank you for taking the time to read the manuscript again and consider my paper.

Corresponding author, Jie Men, menjie2020@126.com，+86-18903588568.
