# Supplementary material for: Effects of high-intensity interval training on physical morphology, cardiorespiratory fitness and metabolic risk factors of cardiovascular disease in children and adolescents: A systematic review and meta-analysis
Source: PLoS One. 2023 May 11;18(5):e0271845. doi: 10.1371/journal.pone.0271845 (PMC10174557; doi:10.1371/journal.pone.0271845)
Supplement: Attachment — Submitted filename: Response to Reviewers.docx [file pone.0271845.s034.docx]

Dear editors and reviewers，

Thank you for the opportunity to improve the quality of our manuscript (PONE-D-22-19095). We appreciate the reviewer’s constructive and insightful comment. We have again searched the literature and made necessary revisions in response to the editor's suggestions. We hope that the revised manuscript has now met the publication standard of your journal. All the changes are highlighted in green to distinguish them from the first changes made in red.

After searching the PubMed MeSH database, we determined that all expressions related to “cardiorespiratory fitness”/ “cardiopulmonary fitness” in the text were corrected to “cardiorespiratory fitness”.

After reviewing the literature again, we found that it is not appropriate to simply use lipid metabolism indicators (TC, HDL-C, LDL-C), blood glucose concentration, hypertension, etc. as cardiometabolic indicators. Because the term "cardiometabolic index" is used incorrectly in the text, combining the extensive literature evidence: the most common cardiometabolic risk factors in the population include altered lipid profiles (TC, TG, HDL-C), altered blood pressure, and obesity. Furthermore, these factors tend to cluster together and often occur in combination in individuals [1,2,3], Therefore, we changed the term "cardiometabolic indicators" to "cardiometabolic risk factors," Rationalizes the use of lipid profiles, blood glucose concentrations, and hypertension as biomarkers of cardiovascular disease (i.e., outcome indicators).

After discussion, we found that our age subgroup analysis had some limitations(see the description of the limitations of the discussion). Previously, we also received this revision from you, tried what you said about dividing the covariate of age into at least three groups, but based on the basic characteristics of our included literature - there were only four relevant studies in which the age of the study subjects belonged to young adults - and the large age span of the subject groups themselves in most of the included studies (which could not be clearly grouped) made the sample size of some subgroups small enough to support the results of a robust subgroup analysis. Furthermore, differences in participant characteristics between pre-pubertal and pubertal studies with regard to baseline body mass or body composition and health status may confound the interpretation of inter-study differences and their attribution to maturity per se[3,4]. Therefore, few similar meta-analyses in the literature would use maturity (prepubertal and postpubertal) as a covariate for subgroup analysis, let alone for detailed grouping. The subgroup we provide as age, although broad in scope, also allows for a more comprehensive analysis of significant influences on heterogeneity with some credibility.

I hope you are satisfied with our changes. If you have any questions, please be sure to let me know, I will reply to you as soon as possible after receiving comments.

Declaration: The manuscript (in whole or part) has not been submitted or published in other journals at the same time. All authors have read and agreed to the content of the manuscript, and there is no potential conflict of interest.

Thank you for taking the time to read the manuscript again and consider my paper.

Corresponding author, Jie Men, menjie2020@126.com，+86-18903588568.

**Reference:**

1. Monserrat Solera-Martínez ÁHIM. High-Intensity Interval Training and Cardiometabolic Risk Factors in Children: A Meta-analysis. PEDIATRICS. 2021;148(4):e2021050810.
2. Liu J, Zhu L, Su Y. Comparative Effectiveness of High-Intensity Interval Training and Moderate-Intensity Continuous Training for Cardiometabolic Risk Factors and Cardiorespiratory Fitness in Childhood Obesity: A Meta-Analysis of Randomized Controlled Trials. FRONT PHYSIOL. 2020 2020-04-03;11.
3. Weston KL, Azevedo LB, Bock S, Weston M, George KP, Batterham AM. Effect of Novel, School-Based High-Intensity Interval Training (HIT) on Cardiometabolic Health in Adolescents: Project FFAB (Fun Fast Activity Blasts) - An Exploratory Controlled Before-And-After Trial. PLOS ONE. 2016 2016-08-03;11(8):e159116.
4. Eddolls WTB, McNarry MA, Stratton G, Winn CON, Mackintosh KA. High-Intensity Interval Training Interventions in Children and Adolescents: A Systematic Review. SPORTS MED. 2017;47(11):2363-74.
