# Supplementary material for: Effects of high-intensity interval training on physical morphology, cardiorespiratory fitness and metabolic risk factors of cardiovascular disease in children and adolescents: A systematic review and meta-analysis
Source: PLoS One. 2023 May 11;18(5):e0271845. doi: 10.1371/journal.pone.0271845 (PMC10174557; doi:10.1371/journal.pone.0271845)
Supplement: Attachment — Submitted filename: Response to Reviewers.docx [file pone.0271845.s035.docx]

Dear Editors and Reviewers,

Thank you for providing us with the opportunity to improve the quality of your submissions (PONE-D-22-19095). We appreciate the constructive and insightful comments from the reviewers. In this revision, we have addressed all comments and suggestions. We hope that the revised manuscript has now met your publication's publishing standards. We have highlighted all revisions in yellow to distinguish the previous two revisions.

First of all, we carefully read the reviewers' comments, and carefully read and analyzed the problematic literature you raised based on the review comments.

According to the inclusion exclusion criteria for this paper, "Participants: Children and adolescents aged 5-19 years (normal weight, obesity, disease, etc.)." The above literature met the exclusion criteria. Six papers presented by the reviewers: Rosenkranz et al.[43] (experimental group: 8.8±0.6; control group: 9.8±4.1), Mazurek et al.[37] (experimental group: 19.5±0.6; control group: 19.5±0.6), Peter Riis Hansen et al.[41] (experimental group: 8-12 ; control group: 8-12), Ferrete et al.[39] (experimental group: 9.32±0.25; control group: 8.26±0.33), G. Baquet et al.[51] (experimental group:9.7± 0.9; control group: 10.1±0.4), Lambrick et al.[30] (experimental group:9.3± 0.8/9.2±0.7; Control Group: 9.4±0.8/9.2±0.8), all within the age range we included in the exclusion criteria. To assess the effects of H IIT more objectively, we did not limit participants.

We evaluated the effects of HIIT in children and adolescents with the aim of scientifically and objectively reflecting the effects of HIIT on them. In addition, our age subgroup analysis of children and adolescents yielded robust results suggesting that the effects of HIIT on children and adolescents are widespread.

In response to "There is a missing dot in table 2 in the age information: Lambrick et al.[30] 2016 Obesity: 93±0.8 Normal: 92±0.7", We have made corresponding changes in the original text and highlighted the changes.

The above is our response to the reviewers' comments, if we have a deviation in the understanding of the reviewers' comments, or if you have any questions, we look forward to your further replies to deepen our understanding of the reviewers' comments and improve the quality of the paper.

Declaration: The manuscript (in whole or part) has not been submitted or published in other journals at the same time. All authors have read and agreed to the content of the manuscript, and there is no potential conflict of interest.

Thank you for taking the time to read the manuscript again and consider my paper.

Corresponding author, Jie Men, menjie2020@126.com，+86-18903588568.
